# Integrating Historical Learning and Multi-View Attention with Hierarchical Feature Fusion for Robotic Manipulation

**DOI:** 10.3390/biomimetics9110712

**Published:** 2024-11-20

**Authors:** Gaoxiong Lu, Zeyu Yan, Jianing Luo, Wei Li

**Affiliations:** The Academy for Engineering and Technology, Fudan University, Shanghai 200433, China; gxlu22@m.fudan.edu.cn (G.L.); zeyuyan22@m.fudan.edu.cn (Z.Y.); jnluo22@m.fudan.edu.cn (J.L.)

**Keywords:** robotic manipulation, historical information, multi-view attention, hierarchical visual representations

## Abstract

Humans typically make decisions based on past experiences and observations, while in the field of robotic manipulation, the robot’s action prediction often relies solely on current observations, which tends to make robots overlook environmental changes or become ineffective when current observations are suboptimal. To address this pivotal challenge in robotics, inspired by human cognitive processes, we propose our method which integrates historical learning and multi-view attention to improve the performance of robotic manipulation. Based on a spatio-temporal attention mechanism, our method not only combines observations from current and past steps but also integrates historical actions to better perceive changes in robots’ behaviours and their impacts on the environment. We also employ a mutual information-based multi-view attention module to automatically focus on valuable perspectives, thereby incorporating more effective information for decision-making. Furthermore, inspired by human visual system which processes both global context and local texture details, we have devised a method that merges semantic and texture features, aiding robots in understanding the task and enhancing their capability to handle fine-grained tasks. Extensive experiments in RLBench and real-world scenarios demonstrate that our method effectively handles various tasks and exhibits notable robustness and adaptability.

## 1. Introduction

Although learning from demonstration (LfD) has achieved impressive success in robotic domains [1,2], learning a language-guided manipulation policy to predict 3D end-effector pose from visual observations is significantly challenging. On the one hand, imitating human demonstrations for some complex manipulation tasks, e.g., making a coffee, typically involves a line of integrated sub-tasks executed in a sequential manner, which can be formulated as a long-horizon Markov Decision Process (MDP). These tasks often require not only the understanding of abstract language instructions but also the ability to perform a variety of fundamental behaviours that collectively contribute to the completion of the overall manipulation tasks. Unfortunately, conventional imitation learning methods [3], such as behaviour cloning [4], often fall into the dilemma of cumulative compounding errors, leading to a catastrophic performance decline when encountering long-horizon action sequences. To address the challenges in long-horizon tasks, some researches [5,6] applied skill learning to decompose these tasks into sub-tasks. Task planning algorithms subsequently combine these sub-tasks to form a long-horizon task. However, such methods are not end-to-end and require prior collection of relevant skills based on the specific scenario. The limited robustness of individual skills can reduce the overall performance in long-horizon tasks. Robotic manipulation based on LLM [7,8,9,10] have developed rapidly, where object detection techniques first extract objects from the scene, and then task-specific prompts are sent to large language models like GPT-4 [11] to control the robot. Such approaches rely on the accuracy of object detection and the reasoning capabilities of large language models. LLMs may generate hallucinations [12] in many scenarios, resulting in unpredictable behaviors. Our method addresses long-horizon tasks using an end-to-end approach, employing a spatio-temporal attention-based network to fuse historical information, thus enhancing performance in long-horizon tasks. On the other hand, due to the inherently partial perceptibility of visual observations, relying on RGB images from a specific viewpoint exclusively can lead to the omission of critical information, potentially causing significant deviations in executed actions. For instance, when the handle of a drawer is occluded by a robot arm within the camera’s view field, the agent often struggles to rapidly locate the position of core parts, leading to aberrant movements. Although multi-view systems mitigate this by combining multiple viewpoints, they also present challenges. Occlusions from the robot or environment create blind spots [13], requiring intelligent fusion methods to minimize information loss. Additionally, the need to dynamically prioritize the most informative viewpoints for each task is crucial but often missing in traditional static-weighting methods [14]. Moreover, multi-view processing incurs high computational and memory demands because redundant data accumulates across views.

To better encode spatial occlusions and improve spatial reasoning, some appro- aches [15,16] have integrated 3D perceptual representations derived from point clouds, which enhance spatial precision in end-effector pose prediction by providing depth and structural information that 2D images alone cannot offer. However, these methods typically rely on unstructured point cloud data, which is challenging to process directly due to its irregular nature. To address this, manually defined grids or voxelization strategies [17,18] are often applied to transform the point clouds into high-resolution 3D feature representations. While effective in capturing spatial details, these transformations are computationally intensive, particularly at high resolutions, leading to increased memory demands and processing time.

Recently, several approaches [19,20] have advanced the concept of universal representations, leveraging vision models pre-trained on extensive, diverse real-world data to improve semantic feature extraction and provide robots with a broader understanding of task contexts. By capturing a rich array of real-world features, these universal models enhance the robot’s capacity to interpret scene semantics, ultimately enabling better-informed decision-making and task comprehension. However, a notable limitation of these pre-trained models lies in their reduced adaptability to highly specialized or intricate operational settings, such as precision assembly tasks involving fine-grained components like screws and electronic connectors. In these scenarios, the universal representations may lack the detail and context required for precise manipulation.

In response to these limitations, we propose a novel framework for robotic manipulation that emphasizes the integration of historical information, hierarchical feature fusion, and multi-view attention mechanisms. Our contributions are as follows:

Firstly, we incorporate a spatio-temporal attention mechanism that fuses temporal information with the current state, allowing the model to leverage previous actions and observations to enhance decision-making. By incorporating visual observations from previous time steps, the robot can perceive dynamic changes in the environment. Projections of past actions allow the robot to recognize its own action trajectory, which enables self-correction of its behaviour and helps to mitigate the effects of accumulated errors.

Secondly, we introduce a hierarchical feature fusion mechanism that combines global semantic features with local texture details from multi-modal input, such as RGB images and point clouds. This fusion allows the robot to extract global semantic features for task understanding while simultaneously focusing on fine-grained object details necessary for precision manipulation.

**Figure 1:**
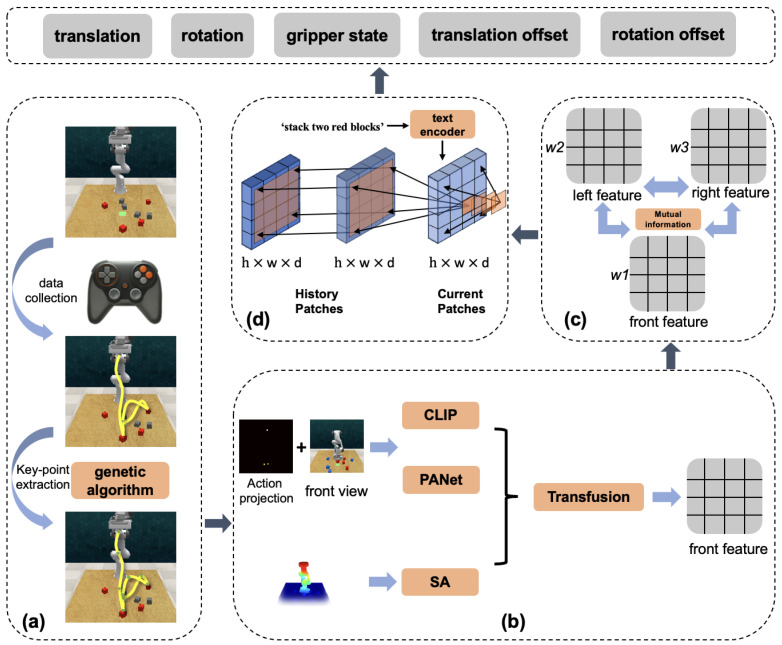
Part (**a**) is the trajectory processing modules. Demonstrations are manually collected using a gamepad, and then macro steps are extracted based on keypoint analysis and genetic algorithms. Part (**b**) extract the hierarchical feature from visual inputs and fuse them by transfusion. The fused visual feature are then processed in the part (**c**), using mutual information to reduce visual feature redundancy and calculate the weight of each viewpoint. Then the multi-view information is weighted and fused. In part (**d**), the fused multi-view features are passed through a spatio-temporal attention network, which then output the actions for the robot to execute. The output actions are composed of the 3D pose of the end-effector, positional offsets and gripper state.

Thirdly, to mitigate the limitations of single-view and traditional multi-view visual input, we propose a mutual information-based multi-view attention mechanism that dynamically allocate weights for different cameras to emphasize viewpoints containing more informative features. In addition, during the action output phase based on 3D point cloud data, we prioritize the viewpoint that contains the most valuable information for more accurate prediction. The pipeline is shown in Figure 1.

## 2. Related Work

### 2.1. Language Conditioned Multi-Task Manipulation

In robotic manipulation, learning-based methods [21] have emerged as powerful tools, particularly in dynamic environments where traditional visual servoing techniques [22] fall short. Multi-task manipulation has gained increasing attention with methods like meta-learning [23], reinforcement learning [24], and imitation learning. These methods often train on various tasks simultaneously, facilitating knowledge transfer and forming a general model. Language instructions play a crucial role in guiding agents to comprehend task requirements and differentiate between tasks. The rise of large language models, such as CLIP [25] and Bert [26], has significantly influenced natural language processing, enabling more effective feature extraction from language instructions. For benchmarking, we chose RLBench [27] and utilized its built-in demonstration generation function, alongside designing language commands for each task.

### 2.2. Visual Representations for Manipulation

Understanding environmental information is critical in robotic manipulation. Visual representations can be categorized into 2D and 3D types, each offering unique benefits: 2D representations, including PANet [28], UNet [29] and ResNet [30], provide rich semantic and texture features, while 3D representations, including PointNet++ [31], C2FARM [32] and PERACT [33] offer comprehensive structural information. In the field of robotic manipulation, pre-trained visual models have recently become a hot topic. These models leverage extensive datasets from both real-world and simulated environments to acquire generic features, thereby supporting a wide range of downstream tasks and significantly saving time and resources. Examples of such models include CLIP [25], R3M [20], and SGR [34]. Our study utilizes a pre-trained visual language model for global semantic feature extraction and a hierarchical feature extraction network for local texture feature extraction, as well as integrating 2D visual features with 3D structural information for action prediction.

### 2.3. Robotic Transformers

The Transformer [35] architecture has achieved significant advancements in natural language processing, computer vision, and robotic manipulation. Its application in robotics extends to diverse areas such as legged locomotion [36], path planning [37] and vision-language navigation [38]. The versatility of the Transformer underscores its ability to tackle intricate robotic tasks, showcasing its adaptability and effectiveness in diverse scenarios. While several methods based on the Transformer have emerged, they seldom fully leverage the remarkable ability of the Transformer to utilize historical data for enhancing action prediction in complex, multi-modal scenarios. PERACT utilizes the Perceiver Transformer to predict actions based on current voxel observation, achieving greater efficiency and robustness. Gato [39] is an example of a multi-modal, multi-task, general-purpose agent. However, Gato relies heavily on large datasets, such as 15 K episodes for block stacking and 94 K episodes for Metaworld tasks. In contrast, our method only requires 50 to 100 demonstrations to complete common tasks.

### 2.4. Multi-View Robotic Manipulation

Multi-view robotic manipulation has garnered significant attention due to its ability to offer richer visual information, leading to more precise and robust manipulation tasks. Using multiple viewpoints helps mitigate occlusions, enhances perception, and increases the accuracy of robotic actions, particularly in complex and cluttered environments. Multiple approaches have been proposed to exploit multi-view setups to improve scene understanding and manipulation precision. Xie and Song [40] proposed a multi-view registration method for partially overlapping point clouds, which is critical for accurate 3D reconstruction in robotic manipulation. Their point-to-plane registration model minimizes cumulative errors in multi-view registration using pose graphs. This method enhances the ability to handle occlusions and noisy data, enabling accurate object handling and placement in complex environments. Lin et al. [41] introduced a multi-view fusion framework for multi-level robotic scene understanding. Their system integrates 2D RGB images and 3D point clouds to create a rich scene representation for robotic manipulation tasks. By combining dense 3D reconstruction for obstacle avoidance, primitive shape fitting for unknown objects, and full 6-DoF object pose estimation for known objects, their approach enhances tasks such as grasping and object rearrangement. Seo et al. [42] presented a novel multi-view masked autoencoder that learns to reconstruct masked pixels from random viewpoints, significantly improving the robot’s perception capabilities. This technique captures both intra-view and cross-view information, improving multi-view control and real-robot task transfer without requiring camera calibration. The work by Song et al. [43] explores learning precise 3D manipulation from multiple uncalibrated cameras. By leveraging camera configurations that do not require pre-calibration, their method simplifies the process of multi-view integration, enhancing manipulation precision through a learning-based approach that directly optimizes task performance. We introduce a mutual information-based attention mechanism that dynamically selects and emphasizes the most informative viewpoints, reducing redundancy and ensuring that the robot’s actions are based on a comprehensive yet efficient representation of the environment.

## 3. Method

### 3.1. Problem Definition

Our proposed method aims to develop a multi-modal, multi-view, history-sensitive strategic framework, denoted as $\pi (a_{t+1}|\left\{l_{h}\right\}{|}_{h=1}^{m},{\left\{o_{i}\right\}}_{i=t-s}^{t},{\left\{a_{i}\right\}}_{i=t-s}^{t})$. This strategy, π, incorporates historical observations ${\left\{o_{i}\right\}}_{i=t-s}^{t}$, actions ${\left\{a_{i}\right\}}_{i=t-s}^{t}$, and a series of language instructions $\left\{l_{h}\right\}{|}_{h=1}^{m}$. In this context, *m* signifies the count of language instructions allocated for each task, and *t* represents the current step. Additionally, *s* denotes the count of the past steps. Notably, in scenarios where the current step is less than two, strategy π relies solely on the current observation. This stems from the negligible influence of environmental changes at the beginning of a task, making it sufficient to use only current observations.

We aim to output the actions for robot to execute, and the action space is defined by the pose of the end-effector $\left(x_{t},y_{t},z_{t},q_{t}^{\omega },q_{t}^{x},q_{t}^{y},q_{t}^{z}\right)$ and the gripper state $g_{t}$ (either open or closed). The parameters $\left(x_{t},y_{t},z_{t}\right)$ denote the position, while $\left(q_{t}^{\omega },q_{t}^{x},q_{t}^{y},q_{t}^{z}\right)$ specify the orientation in quaternion format.

For each specified task, a comprehensive array of language instructions is prepared. The observation at step *t*, $o_{t}$, encompasses RGB images ${\left\{I_{t}^{k}\right\}}_{k=1}^{K}$ and point cloud data ${\left\{P_{t}^{k}\right\}}_{k=1}^{K}$ from *K* perspectives, where *K* equals three in simulation and two in real-world experiments. Here, $I_{t}^{k}$ and $P_{t}^{k}$ both have three channels, with the dimensions *H* and *W* set to 128 in our simulated experiments. The actions at step *t* include all actions executed at the current and past *s* steps. These actions are visualized by projecting the gripper’s position onto a 2D plane, distinguishing past and future actions via thermal intensity.

**Figure 2:**
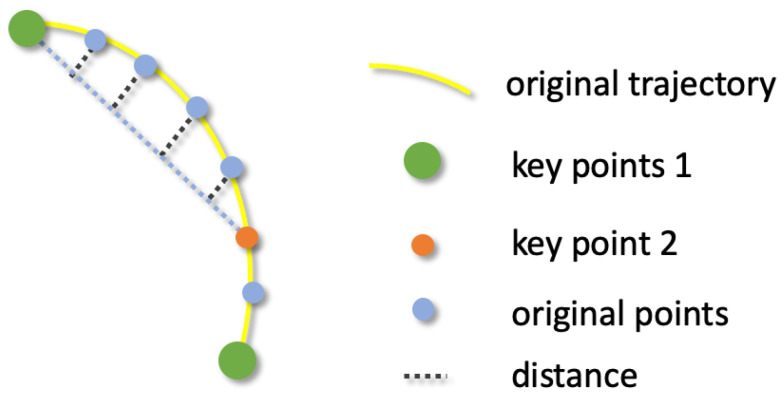
The yellow curve represents the original trajectory, with blue points indicating the original trajectory points. The green points are key points identified by detecting moments when the robotic arm pauses or the gripper state changes. The orange point is a key point selected through the genetic algorithm, which further optimizes the key points to minimize the trajectory error.

As illustrated in Figure 2, our initial step involves the collection of a substantial number of continuous operational trajectories, represented as $T=\{p_{1},p_{2},\ldots ,p_{n}\}$, where $p_{i}$ denotes the position of the end-effector at time step *i*, and *n* is the total number of points in the trajectory. We then refine these trajectories, extracting key steps through a basic method which identifies moments where the end-effector speed is zero or the gripper state alters.

To closely approximate the expert’s trajectory, we employ a genetic algorithm to select other key points. Let $K=\{q_{1},q_{2},\ldots ,q_{k}\}$ be the set of selected key points, where $q_{i}$ corresponds to a subset of the original trajectory points *T*. The number of key points, ranging from 2 to 30, is adaptively selected based on the task’s temporal length. For each non-key point $p_{j}$ between two adjacent key points $q_{i}$ and $q_{i+1}$, we compute the distance from $p_{j}$ to the line segment connecting $q_{i}$ and $q_{i+1}$. This distance is defined as:

(1) $$d\left(p_{j},q_{i},q_{i+1}\right)=\frac{|\left(\vec{q_{i+1}}-\vec{q_{i}}\right)\times \left(\vec{p_{j}}-\vec{q_{i}}\right)|}{∥\vec{q_{i+1}}-\vec{q_{i}}∥}$$

Geometrically, the cross product of $\vec{q_{i+1}}-\vec{q_{i}}$ and $\vec{p_{j}}-\vec{q_{i}}$ gives the magnitude corresponds to the area of the parallelogram, which is proportional to the perpendicular distance between the point $p_{j}$ and the line segment connecting $q_{i}$ and $q_{i+1}$. To convert the area of the parallelogram to the perpendicular distance $d(p_{j},q_{i},q_{i+1})$ (height of the parallelogram), we divide the cross product by the length of the base, which is the distance between the two key points $q_{i}$ and $q_{i+1}$. This gives us the perpendicular distance from $p_{j}$ to the line segment connecting $q_{i}$ and $q_{i+1}$.

The total error $E(T,T^{\prime })$ is the sum of the distances for all non-key points across the entire trajectory:

(2) $$E\left(T,T^{\prime }\right)=\sum_{i=1}^{k-1}\sum_{p_{j}\in S_{i}}d\left(p_{j},q_{i},q_{i+1}\right),$$

where $S_{i}$ is the set of non-key points between $q_{i}$ and $q_{i+1}$.

The optimization goal is to minimize the total error $E(T,T^{\prime })$, ensuring that the simplified trajectory formed by the key points *K* closely approximates the original trajectory *T*. Formally, the optimization problem can be expressed as $\min_{k}E\left(T,T^{\prime }\right)$, where 2≤k≤30 and $E(T,T^{\prime })\leq \epsilon$, *k* denotes the number of extracted key points and ϵ is a predefined error threshold. The genetic algorithm iteratively selects and refines key point sets by optimizing this error function. The fitness of each set of key points is calculated as:

(3) $$\mathrm{fitness}\left(K_{i}\right)=\frac{1}{E(T,T_{i}^{\prime })+\delta }$$

where δ is a small constant to avoid division by zero. The genetic algorithm promotes key point sets that minimize the trajectory error, evolving them over generations until the error is below the threshold ϵ or a maximum number of iterations is reached. In our setup, population size is 100, crossover rate is 0.8, mutation rate is 0.1 and we choose tournament selection as our strategy.

### 3.2. Enhanced Visual Representation

In the ablation study presented in Section 4.5, we observed that relying solely on either CLIP features or PANet for RGB image feature extraction leads to reduced success rates, especially in tasks that demand high precision. For example, the exclusive use of the pre-trained CLIP model significantly lowered the success rate in the inserting peg task. Although these general visual models are effective at capturing the semantic context of the environment and identifying executable tasks, they often lack the resolution needed to recognize fine-grained environmental details critical for precision manipulation.

To overcome this limitation, we propose a novel feature extraction methodology that combines global semantic features with local texture features. This dual-feature design enables the robotic arm not only to comprehend the task but also to perform the precise actions required for its successful execution.

**Figure 3:**
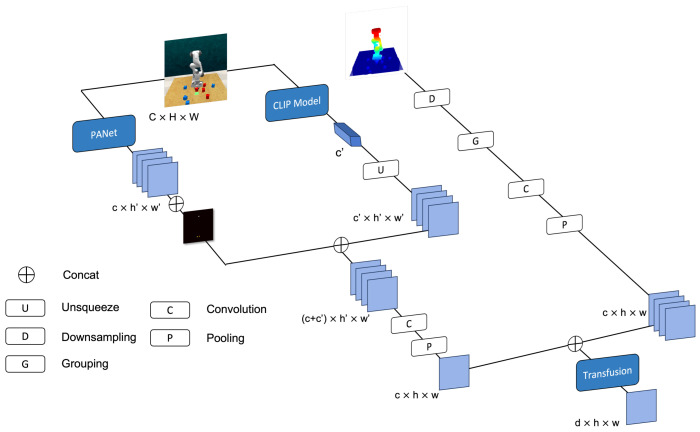
RGB images are processed by both PANet and CLIP models to obtain local texture features (${\mathrm{FR}}_{l}$) and global semantic features (${\mathrm{FR}}_{g}$). These features are combined with the 2D projection of the end-effector pose to form the RGB-A feature (FR). Simultaneously, multi-view point cloud data is processed using the Set Abstraction (SA) module of PointNet++ to extract point cloud features (FP). The fusion of these visual and point cloud features enhances the robot’s ability to interact with complex environments.

As illustrated in Figure 3, for a given viewpoint at certain time step, after the RGB image is processed by both the PANet and CLIP models, we extract the local texture feature (${\mathrm{FR}}_{l}$) and the global semantic feature (${\mathrm{FR}}_{g}$). The local texture feature ${\mathrm{FR}}_{l}$ has dimensions $c^{\prime }\times h^{\prime }\times w^{\prime }$, while the global semantic feature ${\mathrm{FR}}_{g}$ is a one-dimensional vector of length $c^{\prime }$. To incorporate spatial information, we project the end-effector’s pose onto a 2D plane and scale this projection to match the dimensions of ${\mathrm{FR}}_{l}$. We then concatenate the pose projection and ${\mathrm{FR}}_{l}$ along the channel dimension to form the RGB-A feature ${\mathrm{FR}}_{r}$. Subsequently, the global semantic feature ${\mathrm{FR}}_{g}$ is expanded to dimensions $c^{\prime }\times h^{\prime }\times w^{\prime }$, resulting in ${\mathrm{FR}}_{gu}$. Finally, we concatenate ${\mathrm{FR}}_{r}$ and ${\mathrm{FR}}_{gu}$ along the channel dimension, applying convolutional and pooling operations to obtain the final RGB-A feature FR, which effectively integrates both local texture details and global semantic information.

Additionally, we incorporate multi-view point cloud data ${\left\{P^{k}\right\}}_{k=1}^{K}$ into a unified global point cloud dataset *P*. The global point cloud is processed using the Set Abstraction (SA) module from PointNet++, which involves downsampling, grouping, and feature extraction, resulting in a refined set of point cloud features FP. The use of only the SA module for point cloud processing results in significantly lower computational cost compared to other point cloud-based methods. Drawing inspiration from the TransFusion [44] approach, cross-attention is applied to fuse these elements, yielding F=TransFuse(FR,FP), with $F\in R^{d\times h\times w}$.

### 3.3. Mutual Information-Based Multi-View Attention

In multi-view visual tasks, each viewpoint can offer unique information about the environment, but not all views are equally informative. To maximize the use of valuable perspectives, we introduce a mutual information-based attention mechanism that dynamically selects and emphasizes the most informative viewpoints, reducing redundancy and ensuring that the robot’s actions are based on a comprehensive yet efficient representation of the environment.

At certain time step, given the set of feature maps $\left\{F_{1},F_{2},\ldots ,F_{K}\right\}$ extracted from multiple viewpoints, we aim to combine them into a single, representative feature map that retains the most valuable information from each view. However, the challenge lies in ensuring that the fused feature map is both informative and non-redundant. Traditional fusion methods often apply equal or fixed attention to each view, which can result in redundant information, especially when multiple viewpoints capture similar aspects of the scene.

To address this, we propose to use mutual information (MI) as a measure of the shared and unique information between different viewpoints. For two feature maps $F_{i}$ and $F_{j}$, the mutual information $I(F_{i},F_{j})$ quantifies how much information they share. High mutual information indicates redundancy, while low mutual information suggests that the two views contain complementary information. The mutual information between two feature maps is given by:

(4) $$I\left(F_{i},F_{j}\right)=H\left(F_{i}\right)+H\left(F_{j}\right)-H\left(F_{i},F_{j}\right)$$

where $H(F_{i})$ and $H(F_{j})$ represent the entropy of feature maps $F_{i}$ and $F_{j}$, and $H(F_{i},F_{j})$ is their joint entropy. This metric allows us to quantify how much information is shared between different views and use this information to guide the attention mechanism.

To formalize the fusion process, we first compute the mutual information matrix *M*, where each element $M_{ij}$ represents the mutual information between feature maps $F_{i}$ and $F_{j}$:

(5) $$M_{ij}=I\left(F_{i},F_{j}\right)$$

This matrix provides a global overview of the information redundancy across all views. Using this matrix, we then assign dynamic attention weights $\left\{w_{1},w_{2},\ldots ,w_{K}\right\}$ to each view based on the amount of unique information they provide. The weights are learned through optimization, with the objective of minimizing the redundancy in the fused feature map. Specifically, we define the mutual information-based loss function as follows:

(6) $$L_{MI}=-\sum_{i=1}^{K}\sum_{j=1,j\neq i}^{K}w_{i}w_{j}I\left(F_{i},F_{j}\right)$$

In this formula, $I(F_{i},F_{j})$ represents the mutual information between feature maps $F_{i}$ and $F_{j}$, which quantifies the amount of information shared between two views. A high mutual information value indicates that the two feature maps provide redundant information, while a lower value suggests more complementary information.

The weights $w_{i}$ and $w_{j}$ are the attention weights assigned to each view, which are dynamically learned during training. By including the product of these weights in the loss function, the formula aims to penalize pairs of feature maps that have both high mutual information and large attention weights. The intuition behind this is that if two views provide redundant information, their corresponding weights should be reduced. Conversely, views that provide more complementary information will be assigned higher weights.

By minimizing this loss function, we encourage the attention mechanism to assign higher weights to viewpoints that provide unique information while reducing the contribution of redundant viewpoints. The weights are updated dynamically during training through backpropagation, allowing the model to adapt to different scene configurations and viewpoint arrangements.

Once the attention weights are learned, the final fused feature map is computed as a weighted sum of the individual feature maps:

(7) $$\mathrm{FT}=\sum_{i=1}^{K}w_{i}F_{i}$$

This weighted fusion ensures that the final feature map captures the most relevant and complementary information from each viewpoint. By focusing on maximizing the mutual information between views, our attention mechanism reduces redundancy and enhances the robot’s ability to perceive and act in complex environments.

The key advantage of this mutual information-based attention mechanism is its ability to dynamically adapt to the content of the scene and the arrangement of the viewpoints. Unlike traditional approaches that apply equal attention to all views, our method selectively emphasizes the most informative perspectives, resulting in a more efficient and informative representation. This improved representation not only enhances the robot’s perception but also improves its decision-making in tasks that require precise interaction with the environment. The mutual information-based attention mechanism ensures that the robot focuses on the most valuable perspectives, effectively reducing redundancy and maximizing the use of complementary information.

### 3.4. History-Sensitive Decision Network

Initially, we introduce the attention mechanism inherent in the Transformer architecture:

(8) $$\mathrm{Attn}\left(Q,K,V\right)=\mathrm{Softmax}\left(\frac{W_{q}Q{\left(W_{k}K\right)}^{T}}{\sqrt{d}}\right)W_{v}V$$

where $W_{q},W_{k},W_{v}$ represent trainable parameters. In our proposed method, cross-attention is employed to fuse RGB-A features with point cloud data, forming the foundation of a history-sensitive decision network that utilizes self-attention.

Subsequent to the enhanced visual representation(EVR) module and multi-view attention(MVA) module, the integrated feature map at time step *t*, $F_{t}$, effectively segments the initial H×W image into h×w discrete patches. Initially, language instructions processed through the CLIP model undergo text preprocessing, tokenization and embedding. The resulting embeddings are flattened and subsequently incorporated into each patch’s channels, embedding linguistic information into the feature map through a convolutional neural network. Furthermore, both step and patch position encodings are infused into each channel of this feature map, integrating essential temporal and spatial information. Through the design of padding and causal encoding mechanisms, and leveraging the self-attention mechanism of the Transformer, we enable each patch to interact with other patches at the current step as well as those from the past several steps, culminating in a history-enriched feature map $F_{t}\in R^{d\times h\times w}$.

**Figure 4:**
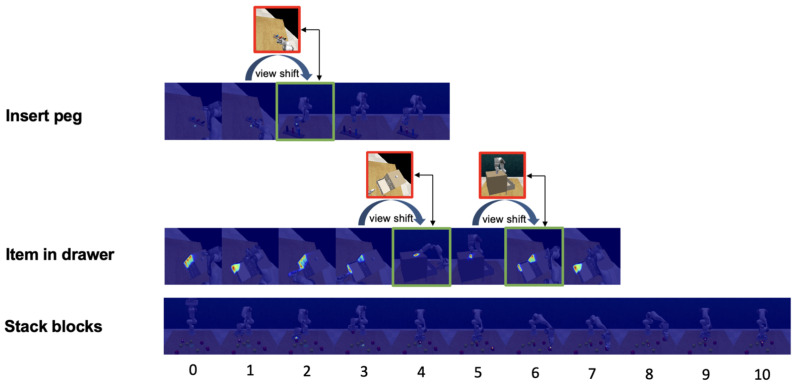
The double-head arrow connects the viewpoints before (red box) and after (green box) the view shift. In the task inserting peg, the perspective shifts from the left shoulder view to the front view at the 2nd step as the robot arm blocks the target object from the left shoulder view. In the task item in drawer, the multi-view attention module considers the front viewpoint more valuable at the 4th and 5th steps. In the task stacking blocks, there are no changes in viewpoint.

$F_{t}$ is then concatenated with multi-level feature maps extracted with PANet and successively upsampled layer by layer, focusing exclusively on the perspective with the most valuable information, to reconstitute the feature map to its original dimensions of d×H×W. As Figure 4 reveals, the position decoder, essentially a convolutional layer with a single output channel, transforms the feature map into a $heatmap\in R^{1\times H\times W}$. When combined with the most informative viewpoint’s point cloud data and aggregated across the channel dimension, the heatmap generates precise position coordinates $\left(x_{t},y_{t},z_{t}\right)$.

Subsequently, $F_{t}$ and ${\mathrm{FR}}_{t}$ undergo concatenation and a decoder, comprising dual convolutional layers, a pooling layer, and a pair of dense layers, produces a seven-dimensional output $\left(xo_{t},yo_{t},zo_{t},q_{t}^{\omega },q_{t}^{x},q_{t}^{y},q_{t}^{z},g_{t}\right)$. Given that point cloud data primarily represents physically present points in a scene, and considering scenarios where the robotic arm is required to access virtual points around an object, the parameters $\left(xo_{t},yo_{t},zo_{t}\right)$ are designated to define positional offsets, thereby enabling the robotic arm to proficiently navigate and reach these virtual spatial points.

### 3.5. Training Details

The training of our model is conducted through behaviour cloning. For each variation of a task, such as opening the middle drawer and opening the bottom drawer being considered as separate entities under the same task, we collect a set of *N* successful trajectories, denoted as *D*. This process involves key point extraction utilizing genetic algorithms, subsequently leading to the identification of macro steps across amounts of steps. Each demonstration, symbolized as δ∈D, is constituted by a succession of these identified macro steps and B contains a batch of demonstrations.

Our final loss function includes position loss, rotation loss, gripper loss, and mutual information loss. The action loss (position, rotation, and gripper losses) ensures the model accurately predicts the robot’s action based on the expert demonstrations, while the mutual information loss ensures that redundant information across multiple views is minimized during feature fusion. The combined loss function is defined as:

(9) $$L=\lambda_{1}\left(\frac{1}{\left|B\right|}\sum_{\delta \in B}\left[\sum_{t\leq T}\left(\mathrm{MSE}\left(pose_{t},pose_{t}^{*}\right)+\mathrm{MSE}\left(gp_{t},gp_{t}^{*}\right)\right)\right]\right)+\lambda_{2}L_{MI},$$

where:

- $pose_{t}$ denotes the predicted pose at time *t* (including position $pos_{t}$ and orientation $rot_{t}$) and $pose_{t}^{*}$ is the real pose in demonstration.
- $gp_{t}$ represents the predicted gripper state (either open or closed) and $gp_{t}^{*}$ is the real gripper state in demonstration.
- $L_{MI}$ is the mutual information loss, as Equation (9) shown, calculated by measuring the mutual information between feature maps from different views. It penalizes redundancy and encourages extracting complementary information from different viewpoints.
- $\lambda_{1}$ and $\lambda_{2}$ are hyperparameters that control the relative importance of the action loss and mutual information loss respectively.

The training procedure is presented in Algorithm 1:
**Algorithm 1** Training Procedure 1:**Input:** Set of successful trajectories *D*, batch size *B*, learning rate α, max training iterations $T_{max}$, loss weights $\lambda_{1}$ and $\lambda_{2}$ 2:**for** each training iteration t=1 to $T_{max}$ **do** 3:   Sample a batch of demonstrations {δ}∈D of size *B* 4:   Initialize total loss L=0 5:   **for** each demonstration δ in batch **do** 6:     **for** each time step *t* in demonstration δ **do** 7:        Extract robot’s target pose $pose_{t}^{*}$ and gripper state $gp_{t}^{*}$ from demonstration 8:        Predict pose ${\mathrm{pose}}_{t}$ and gripper state ${\mathrm{gp}}_{t}$ at time *t* with model ${\mathrm{Model}}_{\theta }$ 9:        Calculate position loss: $L_{pos}$ and gripper loss: $L_{gp}$10:     **end for**11:     Calculate action loss: $L_{action}=\frac{1}{T}\sum_{t=1}^{T}\left(L_{pos}+L_{gp}\right)$12:     Calculate mutual-information loss: $L_{MI}$13:     Total loss: $L_{\delta }=\lambda_{1}\cdot L_{action}+\lambda_{2}\cdot L_{MI}$14:     Accumulate batch loss: $L=L+L_{\delta }$15:   **end for**16:   Update model parameters: $\theta \leftarrow \theta -\alpha \cdot \nabla_{\theta }L$17:**end for**18:**Output** Trained model with parameters θ

## 4. Experiments

To evaluate the efficacy of our proposed method, we conducted a series of experiments encompassing single-task, multi-task, and long-horizon-task experiments. In the single-task experimental setup, a particular variant of the task was considered as an instance of that task. Conversely, within the multi-task experimental framework, different variants of a single task were recognized as distinct and individual tasks. For example, picking up a red cup and picking up a green cup were treated as two separate and independent tasks.

During the training phase, we collected 50 demonstrations for both single-task and long-horizon tasks. The trajectory points in single-task demonstrations are generally fewer than 50, whereas long-horizon tasks have more than 50 trajectory points. In our designed long-horizon tasks, the number of trajectory points exceeds 100. Meanwhile, in order to validate the relationship between the number of demonstrations and multi-task performance, the number of demonstrations for the multi-task training varied, encompassing either 10 or 100 demonstrations. With a batch size of 16 and a learning rate of $5\times 10^{-4}$, the single-task model was trained for 100,000 iterations. In contrast, the training of the multi-task model took 300,000 iterations. During the testing phase, the experiments were conducted under the influence of three distinct random seeds. Each task corresponding to a specific seed underwent 100 testing episodes in all evaluation settings.

### 4.1. Single Task Experiments

We tested 77 RLBench tasks, from which we selected 8 challenging tasks for our single-task experimental analysis, training a distinct model for each task variant. As illustrated in Table 1, our method was benchmarked against PERACT [33], SGR [34], and R3M [20] across all selected tasks. PERACT is a multi-task framework that employs the Perceiver Transformer to process voxelized observations and predict actions. We reran the project’s code for our experiments and used only single-task demonstrations for training. SGR is a general feature extraction method that combines semantic and geometric representation. This method has conducted a large number of experiments on RLBench, and we directly quote its experimental results. In the R3M experiments, the R3M framework was leveraged only for the extraction of global features during the RGB feature extraction phase, while the action prediction component remained unaltered.

The outcomes of these experiments unequivocally demonstrated that our method outperformed others in all 8 tasks, with average success rates of 84.2%. In particular, our method can still perform well (62.5%) in scenarios demanding high-precision operations (e.g., inserting peg task), while other methods perform much worse. In addition, we have compared the runtime of our method with models like R3M, PERACT, and SGR. In our experiments, our model achieved a balance between computational efficiency and task performance, with a processing speed of approximately 5 actions per second during testing. For comparison, PERACT outputs actions at a speed of 3 actions per second, while both R3M and SGR output actions at 2 actions per second. This demonstrates that our method not only provides superior task performance but also operates more efficiently.

### 4.2. Multi Task Experiments

The ultimate goal in robotic manipulation involves equipping robots with the ability to master many diverse skills simultaneously rather than training a separate strategy for each task, which would be time-consuming and resource-intensive. In light of this, we formulated three distinct sets of multi-task experiments. As revealed in Table 2, each set encompassed a blend of both complex and simple tasks, with each task having up to three variations. In the multi-task setup, each of the three task sets includes both simple and complex tasks. For example, set 1 contains tasks such as turning a tap (a relatively simple task) and stacking cups (a more complex task). By designing the task combinations in this way, we can evaluate the model’s robustness across tasks of varying difficulty levels.

To validate the influence of the number of demonstrations on the robotic performance, we established two demonstration groups, one with 10 and the other with 100 demonstrations. Subsequently, we benchmarked the outcomes against those obtained using PERACT. The findings revealed that performances in multi-task settings generally perform worse than those in single-task settings, a trend that is currently prevalent in multi-task learning because our multi-task training approach merely combined the training data from different tasks without delving into the intricacies of inter-task correlations and knowledge transfer. This problem is vital for the development of multi-task learning and is earmarked for our future research. Nonetheless, our method demonstrated superior performance in multi-task scenarios, surpassing a 60% success rate in all tasks when trained with 100 demonstrations.

### 4.3. Long Horizon Task

Long-horizon tasks represent a formidable challenge within the field of robotic manipulation. Numerous studies have sought to augment robots’ capabilities in managing such tasks by utilizing strategies like task hierarchy and skill learning, an endeavour that aligns with our research objectives. To this end, we strategically utilized basic tasks from RLBench to construct three intricate long-horizon task scenarios. These scenarios were designed to emulate diverse environments, including home (water plants + open door + move hanger), kitchen (take lid + turn tap + open microwave), and assembly (insert peg + insert usb + shape sorter) scenarios. Our method consistently achieved a success rate exceeding 60% across all three scenarios when the model was trained for 300,000 iterations. The performance shows our method’s robust capability to handle the complexities inherent in long-horizon tasks.

### 4.4. Unseen Scenarios

**Figure 5:**
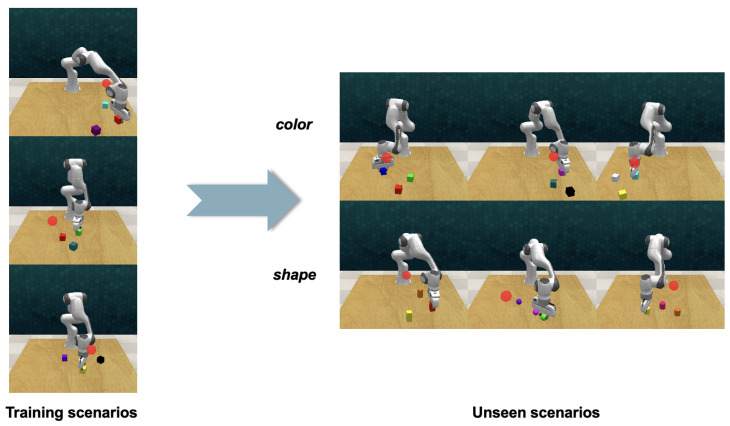
During the testing phase, experiments are conducted with colors and shapes that were not presented during the training phase based on the picking and lifting task.

In order to evaluate the adaptability of our method to scenarios beyond the scope of its training, we conducted some experiments using the picking and lifting task as a test case. This is illustrated in Figure 5. During the testing phase, we introduced block colors and shapes that did not appear in the training phase. We established two demonstration groups, one with 10 and the other with 100 demonstrations. We tested 3 distinct colors and 3 unique shapes, with each variation undergoing 100 testing episodes. The findings (shown in Table 3) were noteworthy: when the robot encountered colors that were unseen from the training dataset, the integration of corresponding language instructions enabled it to achieve a measurable level of success. The outcome not only demonstrates the adaptability of our method but also opens up a promising avenue for future exploration in the realm of robotic manipulation.

### 4.5. Ablation Experiments

In this section, we evaluated the effect of multi-view attention, historical information, and semantic-texture feature extraction of the proposed method.

Table 4 presents the ablation study for each component. Experimental analysis was executed on 30 distinct tasks, each subjected to three unique random seeds.

From the results, we can draw some key conclusions. Utilizing only the historical information module results in a success rate of 47.8% with 100 trajectories. However, when combined with the multi-view attention mechanism, the success rate significantly increases to 71.8%. In contrast, the combination of historical information with the hierarchical feature fusion module achieves a success rate of 68.5%. These findings suggest that the spatio-temporal attention-based action decision network, when integrated with the multi-view attention mechanism, enhances the system’s capability to adapt to environmental changes. Additionally, it is evident that each of the three modules plays an indispensable role in achieving the final performance.

### 4.6. Evaluation on Real Robot

**Figure 6:**
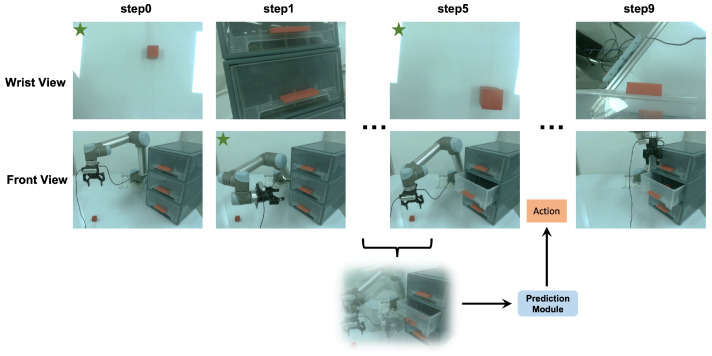
We designed two viewpoints using front and wrist cameras. The viewpoint marked with a green star in the diagram indicates the viewpoint that contains more valuable information. Additionally, the action prediction at each step is based on the observations at the current step, as well as the observations and actions from the past several steps.

To validate the effectiveness of our method in real-world scenarios, we conducted experiments using the UR5 robotic arm. Figure 6 illustrates the experimental setup for one episode of the item in drawer task in the real world. Two RealSense D435 cameras (Intel Corporation, Santa Clara, CA, USA) were positioned to capture different viewpoints, which were fused based on their values computed via multi-view attention. The final action was then determined using point cloud data from the most informative viewpoint. To deal with varying light conditions, which affected the quality of the visual data captured by the RealSense D435 cameras, we collected demonstrations under different lighting scenarios and adjusted the exposure settings dynamically during testing. We also applied noise filtering techniques to the raw point cloud data to remove outliers and reduce the impact of sensor noise. Besides, we used data augmentation techniques such as adding simulated noise to make the model more robust to real-world variations. Additionally, to ensure the safety of the experiments, we restricted the operational range of the robotic arm to a confined space and slowed down the execution speed of the arm’s movements.

For each task, we gathered 50 demonstrations, incorporating data from both the robot’s wrist and front camera to facilitate multi-view analysis. The experiments considered both single-task and multi-task scenarios. We tested each task for 100 episodes. The results (shown in Table 5) indicated that for single-task setting, the overall success rate exceeded 65%. For multi-task setting, although there was a decline in performance, the overall success rate remained above 50%. In the experimental procedure, environmental illumination and the accuracy of point clouds captured by the camera are pivotal factors influencing the success rate.

## 5. Conclusions

In this work, inspired by human cognitive processes and human visual system, we introduce a history-sensitive method that integrates multi-view information and multi-modal inputs. Our approach leverages a spatio-temporal attention mechanism to effectively combine historical observations with current visual data, enhancing the robot’s decision-making capabilities. Additionally, we incorporate a mutual information-based multi-view attention module to dynamically focus on the most informative perspectives, and a hierarchical feature fusion mechanism to merge global semantic features with local texture details. Extensive experiments were conducted in both simulated and real-world environments. The results demonstrate that our method performs well in tasks with varying sequence lengths and exhibits notable robustness and adaptability in unseen scenarios.

However, several limitations remain, which offer directions for future research. For example, the convergence speed during our training is suboptimal. This could be due to the model needing to handle the varying complexities of multiple tasks simultaneously, along with differences in data distribution for each task. The added complexity and variability make the learning process more challenging, thereby slowing down the overall convergence. Also, the performance in real-world scenarios heavily relies on the accuracy of the point cloud data. In environments with varying lighting conditions or occlusions, the precision of the point cloud can degrade, impacting the robot’s decision-making and action execution. In addition, although our method shows adaptability to unseen scenarios, it may struggle with highly complex tasks that require intricate reasoning or long-term planning beyond the current framework’s capabilities. To address the above issues, our future work could focus on developing advanced optimization strategies, such as curriculum learning or meta-learning approaches, to improve convergence rates during multi-task training. To mitigate the dependency on point cloud precision, future research could explore more robust point cloud processing techniques or fusion with other sensory modalities, such as tactile data, to enhance reliability under various environmental conditions. We could also integrate more sophisticated reasoning modules or hierarchical task decomposition strategies to handle more complex tasks. Through these enhancements, we aim to further develop our method and expand its applicability, contributing to the advancement of intelligent robotic manipulation.

## Figures and Tables

**Table 1:** The results of single task.

	Stack Blocks	Item in Drawer	Open Microwave	Water Plants	Toilet Seat Up	Umbrella Out	Unplug Charger	Insert Peg	Average
PERACT [33]	19.0	21.3	34.7	44.3	65.7	90.3	65.3	14.3	44.4
R3M [20]	59.3	55.3	67.0	65.7	81.3	92.3	88.7	31.3	67.6
SGR [34]	-	-	52.6	40.2	80.1	94.7	-	-	-
OURS	88.3	73.7	92.7	78.3	88.0	95.7	97.0	62.3	84.2

**Table 2:** The results of multi tasks (Bold indicates better performance).

Method	Task Set 1 (%)							
	empty dishwasher		knife on board		turn tap		stack cups	
	10	100	10	100	10	100	10	100
PERACT	24.7	41.7	**57.0**	59.3	**77.7**	**93.0**	7.1	10.3
OURS	**32.0**	**74.3**	44.3	**71.7**	72.7	90.3	**28.1**	**68.0**
	**Task Set 2 (%)**							
	**item out of drawer**		**unplug charger**		**water plants**		**usb in computer**	
	**10**	**100**	**10**	**100**	**10**	**100**	**10**	**100**
PERACT	19.9	69.8	47.7	77.0	8.7	45.3	38.0	**77.7**
OURS	**35.4**	**77.7**	**53.3**	**92.7**	**32.3**	**70.7**	**62.3**	76.7
	**Task Set 3 (%)**							
	**stack blocks**		**push buttons**		**reach and drag**		**slide block**	
	**10**	**100**	**10**	**100**	**10**	**100**	**10**	**100**
PERACT	8.1	26.4	35.6	71.3	10.6	40.8	18.0	55.7
OURS	**33.9**	**66.3**	**59.6**	**87.7**	**35.6**	**67.9**	**44.3**	**84.7**

**Table 3:** The results of unseen scenarios.

Demos	Seen Scenarios (%)	Unseen Scenarios (%)	
		Colors	Shapes
10	78.3	39.0	49.7
100	98.7	61.7	81.3

**Table 4:** The results of ablation experiments (Bold indicates best performance).

Multi-View Attention	Historical Information	S-T Feature	Success Rate (%)		
			10	50	100
✓			22.3	23.3	34.2
	✓		28.7	34.5	47.8
		✓	26.4	33.0	35.3
✓	✓		48.7	64.2	71.8
✓		✓	35.4	63.3	77.9
	✓	✓	46.5	59.5	68.5
✓	✓	✓	**69.5**	**70.8**	**82.8**

**Table 5:** The results of real robot experiments.

	Push Button (%)	Item in Drawer (%)	Pick and Lift (%)
Single Task	83	66	77
Multi Task	64	51	71

## Data Availability

The original contributions presented in the study are included in the article, further inquiries can be directed to the corresponding author.
